# Paradox Role of Oxidative Stress in Cancer: State of the Art

**DOI:** 10.3390/antiox11051027

**Published:** 2022-05-23

**Authors:** Cinzia Domenicotti, Barbara Marengo

**Affiliations:** Department of Experimental Medicine, General Pathology Section, University of Genoa, 16132 Genoa, Italy; barbara.marengo@unige.it

The modulation of oxidative stress is essential for the maintenance of redox homeostasis in healthy and cancer cells. In fact, although cancer cells are characterized by the overproduction of reactive oxygen species (ROS) with respect to healthy cells, they are able to survive and proliferate under pro-oxidant conditions by activating redox-sensitive signaling pathways, and by inducing the expression of many antioxidant genes.

Furthermore, oxidative stress can play a fundamental role during all phases of carcinogenesis as well as under therapy-induced stress conditions. In fact, on one hand, ROS over-production contributes to the carcinogenic process by impairing cellular macromolecules and on the other, chemo- and radio-therapeutic agents kill cancer cells via pro-oxidant action. Unfortunately, long-term anticancer therapy has been demonstrated to stimulate antioxidant adaptive responses, contributing to therapy refractoriness.

In addition, antioxidants derived from natural products can exert a chemopreventive action by counteracting cancer development or a chemosensitive effect by potentiating the cytoxicity of anti-cancer therapies. Therefore, focusing the attention on the paradox role of oxidative stress, this Special Issue has collected five research articles, four review articles, and one perspective article that investigates the double-edge role of oxidative stress, which can determine beneficial or detrimental outcomes in cancer development and treatment.

In this context, Nitti et al. have focused their attention on the clinical significance of Heme-oxygenase 1 (HO-1) in cancer progression, and reported contrasting evidence on its role in tumor biology [1]. The authors supported the notion that the pro- or anti-tumor activity of HO-1 is related to its subcellular localization and catalytic activity, and showed a direct correlation between HO-1 over-expression and cancer therapy resistance. Based on the collected findings, they suggest that HO-1 can be a promising biomarker of cancer progression, and in some cases, an interesting target to inhibit in order to increase the cytotoxic effect of standard anti-cancer drugs.

The resistance to anti-cancer therapy is often observed in the clinic, and considering that both iron metabolism and accumulation have been found dysregulated in cancer, the selective induction of ferroptosis could be an alternative anti-cancer strategy. Ferroptosis is an iron-dependent, non-apoptotic regulated cell death characterized by lipid membrane peroxidation and triggered by the depletion of glutathione (GSH), the most important intracellular antioxidant, and by glutathione peroxidase 4 (GPX4) inhibition. In their perspective article, Fujihara and co-authors highlighted that although several studies have described the molecular mechanisms underlying ferroptosis; the clinical use of ferroptosis inducers is limited [2]. However, in this perspective, the authors discussed the possibility to utilize ferroptosis in the clinic (i) by inducing directly ferroptosis in tumor cells and (ii) by lowering the threshold for ferroptosis induction in cancer cells, in order to enhance the efficacy of conventional therapies including chemo, radio, and immunotherapy.

A suggestion in this direction has been given by Monteleone et al., who demonstrated that the combination of etoposide, a known chemotherapeutic drug, with a protein kinase C (PKC)-α inhibitor is able to induce the ferroptosis of cancer stem cells (CSCs) resistant to etoposide [3]. CSCs are characterized by low levels of ROS, high amounts of GSH and over-expression of xCT, a transporter of cystine which is crucial for GSH biosynthesis. Since xCT was found to be stabilized on cell membrane through interaction with CD44, a stemness marker whose expression is modulated by PKC-α, the authors utilized a combined approach of etoposide with sulfasalazine, a direct xCT inhibitor, or with an inhibitor of PKC-α (C2-4). The results obtained demonstrated that the co-treatment with the PKC-α inhibitor was able to induce the ferroptosis of resistant CSCs by inducing aerobic glycolysis, decreasing intracellular GSH levels, down-regulating GPX-4 activity, and stimulating lipid peroxidation.

According to the evidence that the modulation of ROS production might be a valid strategy to counteract tumor development, Menegazzi et al. reported that natural compounds such as *Hypericum perforatum* L. (St. John’s wort, SJW) and its active compound hyperforin (HPF) could exert both a prophylactic and a therapeutic effect [4]. These compounds, by decreasing mitochondrial ROS production and restoring pH imbalance, were able to inhibit cancer cell proliferation, induce apoptosis, down-regulate inflammatory mediators, and inhibit angiogenic factors, limiting tumor growth and spread. The large bioavailability, together with the absence of adverse effects, confer to SJW and HPF a biological relevance for both tumor prevention and treatment, suggesting their potential use in association with current chemotherapeutic drugs.

However, considering other anti-cancer natural compounds, Peng et al. demonstrated that Withaferin (WFA), a triterpenoid isolated from *Whithania somnifera*, combined with a low dose of non-ionizing radiation such as ultraviolet–C (UVC), can act as a therapeutic sensitizer [5]. In fact, low-dose UVC/WFA co-treatment was able to induce ROS over-production, oxidative DNA damage, the inhibition of cell proliferation, and the apoptosis of oral cancer cells without effects on healthy oral cells. Interestingly, this selective therapy deserves in vivo investigations in order to validate the promising in vitro effects of WFA as a UVC chemosensitizer.

Several studies support the notion that the increase in ROS generation is exploited as a valuable anti-cancer strategy. In this regard, Van Loenhout and co-authors focused their attention on therapies based on oxidative stress induced by exogenous pro-oxidant compounds (chemotherapeutic drugs), as well as by targeting endogenous antioxidant molecules [6]. In particular, in this review, the authors provided evidence that ROS-inducing anti-cancer treatments could have a direct effect on tumor microenvironment, exerting both immunostimulatory as well as immunosuppressive effects that must be taken into account during anticancer treatment.

Among ROS-centered anticancer therapies, ionizing radiation (IR) plays a critical role in the management of hematological cancers, even if it is well known that IR is able to impair bone marrow and diverse other organs leading to post-irradiation mortality and morbidity. In this context, Allegra and co-authors reported that an increasing number of compounds able to act as radioprotectors or radiosensitizers has been identified [7]. Among natural and synthetic radioprotective agents, the phytochemical compounds and in particular phenolics (simple phenols, benzoic acid derivatives, flavonoids, stilbenes and tannins) revealed a promising protective effect on healthy cells due to their ability to scavenge ROS production while preserving the sensitivity of cancer cells to IR. On the other side, other phytochemicals such as curcumin, quercetin and genistein have been identified as enhancers of IR treatment or radiosensitizers via oxidative stress induction on cancer cells. In the future perspective to increase the radiosensitivity of cancer cells or to enhance the radioprotection of healthy cells, the authors conclude that patients with lymphomas treated with ultra-high dose rate radiation positively responded to therapy with reduced toxic effects probably due to acute oxygen decrease within the irradiated tissue. Interestingly, it has been demonstrated that miR-139-5p controls the IR therapy response in cancer, and a miR-139-5p mimetic can synergize with IR by increasing ROS production, impairing DNA repair mechanisms and triggering the apoptosis of cancer cells.

Therefore, based on the efficacy of chemotherapy and radiotherapy relying on ROS release, the design of novel ROS modulators could offer new promises for the development of selective and efficient anti-cancer therapies. In this context, Sardella et al. demonstrated that the use of plasma-treated water solutions (PTWS) could be a valid tool able to generate balanced amounts of ROS and reactive nitrogen species (RNS) in liquids [8]. In this study, the authors showed that the synergic action of H_2_O_2_ and NO_2_^−^ can induce the death of osteosarcoma cells, but does not affect endothelial cells of the tumor microenvironment.

Furthermore, Yang et al. described the innovative use of sonodynamic therapy as a sensitizer of ultrasound treatment [9]. They demonstrated that carbon-doped titanium dioxide nanoparticles, made of biocompatible material, are able to inhibit the proliferation of low-intensity ultrasound-treated breast cancer cells through ROS over-production, which sensitizes them to the anti-cancer effect of sonodynamic therapy.

Another approach based on ROS release and used as a chemosensitizer of anticancer therapy is described by Cordani et al. [10]. The authors synthesized triazole-based coordination trimers made with Fe(II) in aqueous media and tested them as adjuvants for the treatment of pancreatic cancer. These coordination complexes were able to stimulate ROS generation in pancreatic cancer cells and enhanced the cytotoxic effect of gemcitabine, an approved drug for pancreatic cancer treatment, through the apoptosis induction and down-regulation of the mTOR pathway.

Altogether, these studies, although in vitro, report different ROS-centered approaches in order to chemosensitize cancer cells to conventional anti-cancer therapies without affecting the viability of healthy cells.

Although many above-described therapeutic strategies need to be tested in animal models, we hope that the articles collected in this Special Issue can clarify the role of ROS modulation in cancer prevention and treatment. Moreover, we believe that the multidisciplinary approach used to address this issue could help to dissect the molecular mechanisms underlying the paradoxical role of oxidative stress in order to counteract carcinogenesis or enhance the sensitivity to anticancer therapy.

We thank all the authors who have contributed to the Special Issue and shared their recent findings, having a potential favorable impact on human health. Finally, we would like to thank the reviewers for their review processing and the Editorial staff for their kind support.
